# *Brassica* and *Sinapis* Seeds in Medieval Archaeological Sites: An Example of Multiproxy Analysis for Their Identification and Ethnobotanical Interpretation

**DOI:** 10.3390/plants11162100

**Published:** 2022-08-12

**Authors:** Giovanna Bosi, Simona De Felice, Michael J. Wilkinson, Joël Allainguillaume, Laura Arru, Juri Nascimbene, Fabrizio Buldrini

**Affiliations:** 1 Department of Life Science, University of Modena and Reggio Emilia, 41125 Modena, Italy; 2 Institute of Biological Environmental and Rural Sciences, Aberystwyth University, Aberystwyth SY23 3EE, UK; 3 Department of Applied Sciences, University of the West of England, Bristol BS16 1QY, UK; 4 BIOME Lab—Department of Biological, Geological and Environmental Sciences, Alma Mater Studiorum University of Bologna, 40126 Bologna, Italy; 5 Sistema Museale di Ateneo, Alma Mater Studiorum University of Bologna, 40126 Bologna, Italy

**Keywords:** Ferrara, Lugo, northern Italy, Middle Ages, Renaissance, archaeobotany, a-DNA, herbaria

## Abstract

The genus *Brassica* includes some of the most important vegetable and oil crops worldwide. Many *Brassica* seeds (which can show diagnostic characters useful for species identification) were recovered from two archaeological sites in northern Italy, dated from between the Middle Ages and the Renaissance. We tested the combined use of archaeobotanical keys, ancient DNA barcoding, and references to ancient herbarium specimens to address the issue of diagnostic uncertainty. An unequivocal conventional diagnosis was possible for much of the material recovered, with the samples dominated by five *Brassica* species and *Sinapis*. The analysis using ancient DNA was restricted to the seeds with a *Brassica*-type structure and deployed a variant of multiplexed tandem PCR. The quality of diagnosis strongly depended on the molecular locus used. Nevertheless, many seeds were diagnosed down to species level, in concordance with their morphological identification, using one primer set from the core barcode site (*matK*). The number of specimens found in the Renaissance herbaria was not high; *Brassica nigra*, which is of great ethnobotanical importance, was the most common taxon. Thus, the combined use of independent means of species identification is particularly important when studying the early use of closely related crops, such as Brassicaceae.

## 1. Introduction

The Brassicaceae are a large family of flowering plants with a circumpolar distribution, comprising 338 genera and 3700 species, including several major agricultural crops [1,2]. The type genus, *Brassica*, is by far the most important economically, and is currently grown in over 150 countries as a vegetable or oilseed crop [3]. Six *Brassica* species dominate current global production, viz.: *Brassica oleracea* L., *Brassica rapa* L., *Brassica napus* L., *Brassica carinata* A. Braun, *Brassica nigra* (L.) K. Koch, and *Brassica juncea* (L.) Czern [4]. Polyploidy has played a key role in the evolution of the cultivation of *Brassica* species, with allopolyploid hybridization between the three diploid species (*B. rapa*, AA, *B. nigra*, BB, and *B. oleracea*, CC) giving rise to three allotetraploids (*B. juncea*, AABB, *B. napus*, AACC, and *B. carinata*, BBCC—[5]). The domestication of these species is thought to have originated around the Mediterranean region of Europe [6,7,8]. The family also contains several genera that feature as minor crops, including (among others): *Raphanus* (*R. sativus* L., the radish), *Armoracia* (*A. rusticana* G. Gaertn., B. Mey. et Scherb., horseradish), *Camelina* (*C. sativa* (L.) Crantz, gold of pleasure), *Nasturtium* (*N. officinale* R. Br., watercress) and *Sinapis* (e.g., *S. alba* L., white mustard). These species have a long history of cultivation, particularly the Brassicas and *Sinapis* [9]. The Italian flora contains 70 genera with 311 species of Brassicaceae, including all of the cultivated species named above [10].

Archeological information can provide useful insights into the timing, context, and nature of domestication events, but first requires appropriate methods for the reliable identification of old, often degraded plant materials. This problem can be addressed by reference to diagnostic phenotypic and molecular information sources. The fruits of Brassicaceae are widely used to perform species diagnosis within the family and can be a useful source of information from archaeological settings when present. Most of the taxa in the family contain siliques and silicle/silicula. The distinction of many of these species is often based on the length/width ratio of the siliques, or on whether they possess bivalved capsules that open from below [11,12]. However, some taxa possess fruit structures of a different kind, such as the lomentum type found in *Raphanus* [13]. Here, too, the features are often useful for the identification of taxa. The seeds are probably the most useful in an archaeological context for diagnostic purposes, and range in shape from spherical to flattened, feature diagnostic sculpturing of the testa, and are often the dispersal unit [11].

European records of Brassicaceae associated with archaeological contexts date back to the Neolithic period, after which they spread geographically, with some records ambiguously listed as weeds s.l., but others with clear evidence of cultivation, primarily as oil crops or vegetables [9,14]. The most frequent and reliable finds for notable economic taxa have been seeds of *Brassica*, *Sinapis*, and *Camelina*, although the certainty of identification can be compromised if the condition of the seeds is poor, especially if they are charred [9]. However, when in good conditions, these features are diagnostic for several taxa [15]. Given the variability in the condition of archaeological materials, there is a need to adopt multiple approaches for species diagnosis, especially when handling partly degraded materials.

Currently, in Italy, more than 700 sites have been investigated from an archaeobotanical viewpoint [16,17]. In the prehistorical period, Brassicaceae seeds were practically irrelevant in the sites of northern Italy [18,19], whereas in a synthesis on the seeds/fruits of food plants for the Roman period in northern Italy, only a few seeds of *Brassica* sp. pl. and *Sinapis alba* were found in 4 sites out of 70 (ca. 6%), and never in funerary-ritual contexts [20,21]. Based on the preliminary data of an analogous synthesis for the Middle Ages/Renaissance period [22], remains of the taxa examined were found in 19 sites out of ca. 50 (about 40%), of which 16 were in the Emilia-Romagna region.

**Figure 1:**
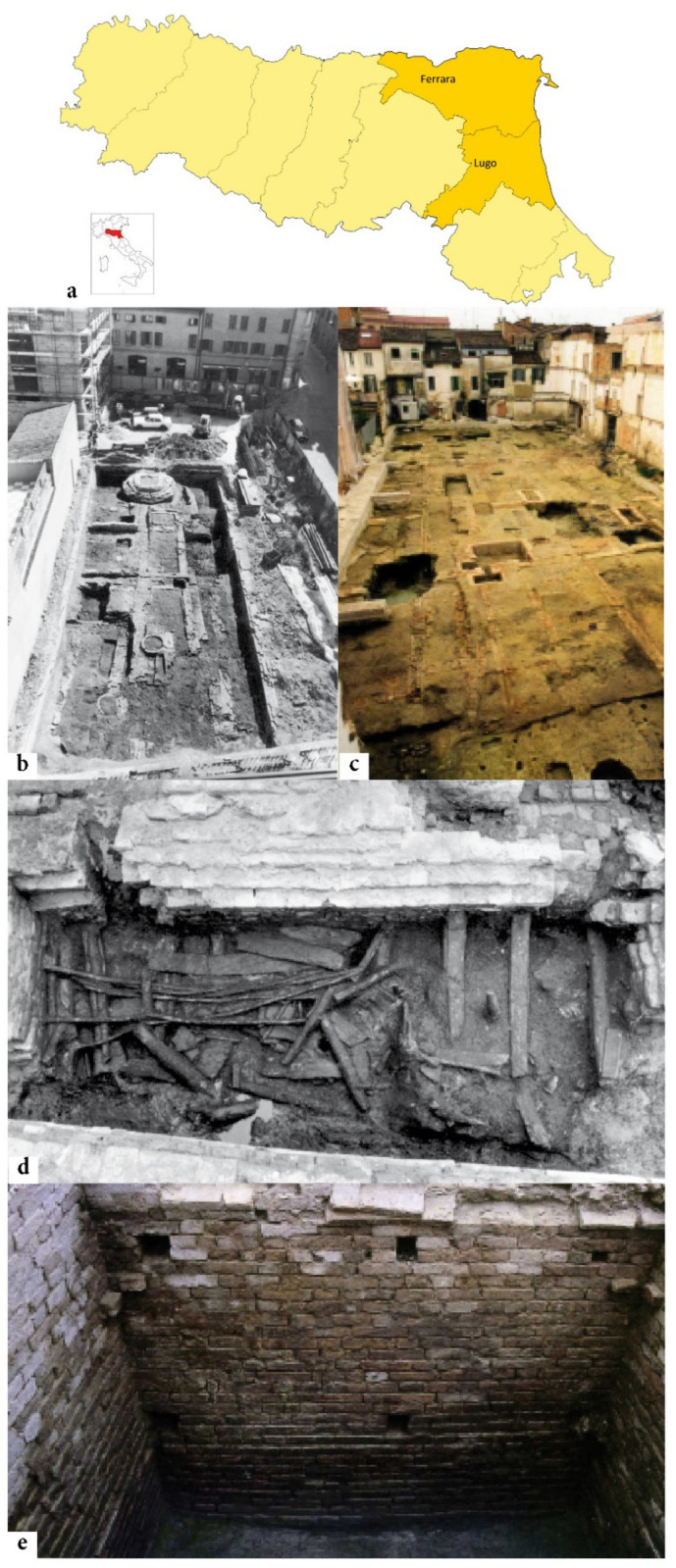
The two archaeological sites examined: Ferrara (context I—no. 16c) and Lugo (context II—no. 15); geographic location (**a**), overview of archaeological excavation (**b**,**c**) and pits (**d**,**e**). Photographs presented with permission from Soprintendenza Archeologia, Belle Arti e Paesaggio Bologna-Modena-Reggio Emilia-Ferrara.

An archaeological site in the historic centre of Ferrara (Emilia-Romagna, Northern Italy—Figure 1) has revealed deposits, datable to between the Middle Ages and the Renaissance, rich in botanical records, which are still being studied. This site contains an unusually large quantity of Brassicaceae seeds (in particular of *Brassica/Sinapis* type), and was therefore deemed likely to present challenges for reliable species identification. Similarly, another coeval site in Lugo (Ravenna, Emilia-Romagna—Figure 1) also returned a good number of *Brassica/Sinapis* seeds and was also considered a suitable site to investigate the fidelity of species assignation.

In this study, the seeds were investigated by morphometric and genetic analyses. Furthermore, a historical and ethnobotanical interpretation of the taxa was performed using written sources and the *exsiccata* present in the oldest Italian herbaria.

## 2. Results

The Emilia-Romagna archaeological sites dating from the 6th to the 17th century AD included 19 contexts in which seeds were found, although in the majority of these (about 58%), the seeds were present at concentrations below 1 seed/L. Furthermore, in the vast majority of the contexts (about 84%), *Brassica* and *Sinapis* seeds/fruits accounted for <1% of all the seeds/fruits recovered. In 7 contexts (nos. 3, 7, 12a, 13, 15, 16a and 16b), the concentration of seeds/fruits varied between 1 and 13 seeds/L, although in all these cases, the Brassicaceae seeds represented ≤1% of all the seeds found. The sole context that showed a notable divergence from these trends was the brick tank in Ferrara (no. 16c—context I in Materials and methods), where the concentration of *Brassica/Sinapis* exceeded 1700 seeds/L and accounted for more than 10% of all the seeds found. An intermediate condition was observed in the coeval context no. 15, where *Brassica/Sinapis* were found in modest concentrations (8 seeds/L) and represented < 1% of all the seeds collected (Table 1).

### 2.1. Archaeobotanical Analysis

The individual plant taxa varied in the distribution of the contexts in which they were found. The most widely found of the taxa identified to species rank were *Rapistrum rugosum* (L.) All. (11 of 19 contexts) and *Brassica rapa* (11 contexts), although the latter was far more abundant throughout. Most of the other species featured only in a minority of the contexts surveyed. For example, *Sinapis alba* seeds were identified with high certainty in several contexts, although few such seeds were found overall (present in 4 of the 19 contexts). The seeds of the genus *Brassica* were more abundant, with well-preserved representatives of 5 distinct species recorded in 11 of the 19 contexts (*B. rapa*, *B. napus*, *B. juncea*, *B. nigra*, and *B. oleracea*). Of these, the vast majority (ca. 95%) were discarded as *B. rapa*, but the residual *Brassica* seeds were far more restricted and subdivided between *B. nigra* (1.6%; 2 contexts), *B. napus* (1.4%; 5 contexts), *B. oleracea* (<1%; 1 context), and *B. juncea* (<1%; 1 context). Most of the other confirmed species were similarly restricted to 5 contexts or less, the sole exception being *Myagrum perfoliatum* L. (8 contexts).

The presence of less well-preserved or abnormal/intermediate samples meant that many of the samples could only be identified at higher taxonomic levels. For instance, around 2.5% of the seeds could only be provisionally identified as either unknown *Brassica* seeds (*Brassica* sp.) or as *Brassica/Sinapis* seeds, and species identity could not be determined for the seeds of the genera *Lepidium* (3 contexts), *Rorippa* (2), or *Sisymbrium* (1); the genus even remained unidentifiable for some seeds.

In addition to *Brassica* sp.pl. and *Sinapis alba*, some other taxa may also have had ethnobotanical value (e.g., *Camelina sativa* and *Isatis tinctoria* L.); others were probably simple instances of weeds s.l.

### 2.2. a-DNA Barcoding

As expected, the ancient seed samples invariably provided DNA yields that were too low and degraded to allow detection by agarose-gel electrophoresis or conventional spectral photometry. Similarly, all attempts using standard barcode primers and protocols used for conventional *rbcL* or *matK* barcoding failed to generate detectable amplicons. The application of the MT-PCR method did nevertheless succeed in producing visible amplicons for both barcoding genes. Success was modest for *rbcL*. The first of the two regions targeted (*rbcL*1) yielded only 8 products of the expected 218 bp, but none of the sequences generated from these amplicons matched either the *Brassica* or the *Sinapis* species expected from the seed morphology. However, 39 seeds produced strong amplification products for the second region (*rbcL*2, 203 bp), and most of these yielded sequences of variable quality in both directions. BLASTn searches of the trimmed sequences against the NCBI database invariably identified the appropriate region of *rbcL*. Subsequent searches of the BOLD Systems database revealed matches with sequence homologies above 95% for all but 8 of the samples (Table 2). Most of the remaining samples matched the species that were clearly incongruent with the seed morphology and were therefore deemed contaminant amplicons. Most of these matched species or genera that were likely to have been in the vicinity of the excavation sites, such as *Citrus*, *Juniperus*, *Pinus*, *Picea* or *Cedrus*. Thus, only nine samples were matched most closely to the three *Brassica* species (*B. rapa*, *B. napus* and *B. oleracea*) in the BOLD Systems database and the reference panel (Table 2).

Greater success was achieved using primers that targeted the *matK* gene. Here, all six loci targeted within the *matK* gene produced sequences that corresponded to one of several members of the Brassicaceae. These are each described below, in turn.

*MatK*1. The different *matK* primers for this amplicon varied in their capacity to generate viable products and in the ability of the resultant sequences to differentiate between species held on public databases. The conventional *matK*1 primers performed the least well. These primers yielded only 12 sequences of modest quality, and none matched significantly with any reference barcode held on either the BOLD Systems or on the NCBI database. The performances of the *matk*1a primers were substantially better, with modest quality sequences of 42–182 bp in length (after trimming) secured from 64 samples. Here, matches were recovered for 41 of the 65 samples. Most of these matched equally well to multiple species in terms of percentage identity, score quality, and e-value, but in all cases, the most strongly matched species were all members of the Brassicaceae family. A finer-level diagnosis than family was not possible for 7 samples, but of the remaining sequences, 16 were assigned to the tribe *Brassiceae*, 10 to the genus *Brassica*, one to another genus in the family, four to a single species of *Brassica*, and three to another species within the family (Supplementary Information Table S1). The *matk*1b primers yielded fewer sequences (26), but these were generally longer (150–212 bp) than the *matk*1a. Again, matches were secured for all 26 using NCBI BLASTn searches (Supplementary Information Table S1). Of these, 14 matched two or more species of *Brassica* (genus-level diagnosis) and six matched a single *Brassica* species. The remaining six samples matched species, genera, or families outside the Brassicaceae and were therefore deemed contaminant sequences. The performance of *matk*1c was slightly improved compared with the other *matK*1 combinations. Trimmed sequences of between 118 and 181 bp were secured from 36 samples. Following BLASTn searches of the NCBI database, 26 species were matched to two or more species of *Brassica* and one was identified to a single *Brassica* species. The remaining samples were identified to the same level as the BOLD systems.

*MatK*4. These primers generated 19 sequences of generally good quality that varied in length between 189 and 279 bp, with 17 sequences falling in the range of 262–279 bp. Searches using these sequences produced matches with both databases (Supplementary Information Table S1). Following sequence-homology searches, all except the shortest sequence matched two or more members of the genus *Brassica* (genus-level diagnosis). The shortest sequence failed to match any species above the 95% similarity threshold.

*Matk*5. This combination of primers generated the most extensive set of sequences (90 samples), which varied in length from 75 to 225 bp. These sequences generated diagnoses at various taxonomic levels when searched against both public databases (Table 3). The finest level of diagnosis was obtained when these sequences were subject to BLASTn searches on the NCBI database. Here, no sequences matched the reference barcodes of a single species. However, the number of hits on the NCBI database was high enough that the frequency of hits to one target frequently allowed the provisional identification of the sample. For example, 11 samples matched equally with 52 reference sequences of *B. oleracea*, but also to four other sequences representing three other species. Given the scope for misidentification and the extent of hybridization in the genus, these 11 sample sequences were therefore designated as provisional *B. oleracea*. Similar frequency distributions were also noted for 17 sample sequences matching *B. nigra* and its allotetraploid sister species, *B. carinata*. However, given that the latter is native to Eastern Africa [40], these samples were provisionally identified as *B. nigra* only. The diploid–allotetraploid sister pairing of *B. napus*/*B. rapa* could not be distinguished using this amplicon, but collectively produced another highly skewed distribution, with 11 sample sequences falling into this category (provisional *B. napus* or *B. rapa*). Thus, samples assigned to *Brassica* species belonging to the cultivated U-triangle group [5] accounted for 43% (39/90) of the sequences retrieved. Among the remainder, a further 7 samples matched multiple *Brassica* species equally (genus level diagnosis). The residual 45 samples (50%) were identified at tribe or family level or with a single species of the family outside the type genus *Brassica*, and were therefore deemed inaccurate diagnoses on the basis of incongruence with seed morphology (species-level diagnosis, Brassicaceae; see Table 3).

### 2.3. Information from Ancient Herbaria

The search for samples of *Brassica* and *Sinapis* produced results only for 3 of the 7 assessed herbaria and made it possible to find 25 specimens (Table 4). The species recognized with certainty were *B. napus*, *B. nigra*, *B. oleracea*, *B. rapa* and *S. alba*; also in addition, some of the specimens were not clearly identifiable, and were therefore doubtfully attributed to the cited species, or simply to *Brassica* sp.

Erbario Aldrovandi provided the best information, with 18 specimens belonging to 4 species (*B. nigra*, *B. oleracea*, *B. rapa* and *S. alba*), which were dated to the period 1551–1586 [41,42,43,44,45]. Erbario ex Cibo B provided 6 specimens and 4 species (*B. napus*, *B. nigra*, *B. oleracea* and *S. alba*), datable at the period 1550–1553 [46,47]. Erbario Cesalpino provided only 1 specimen of *B. nigra*, datable at the period 1555–1563 [48].

**Figure 2:**
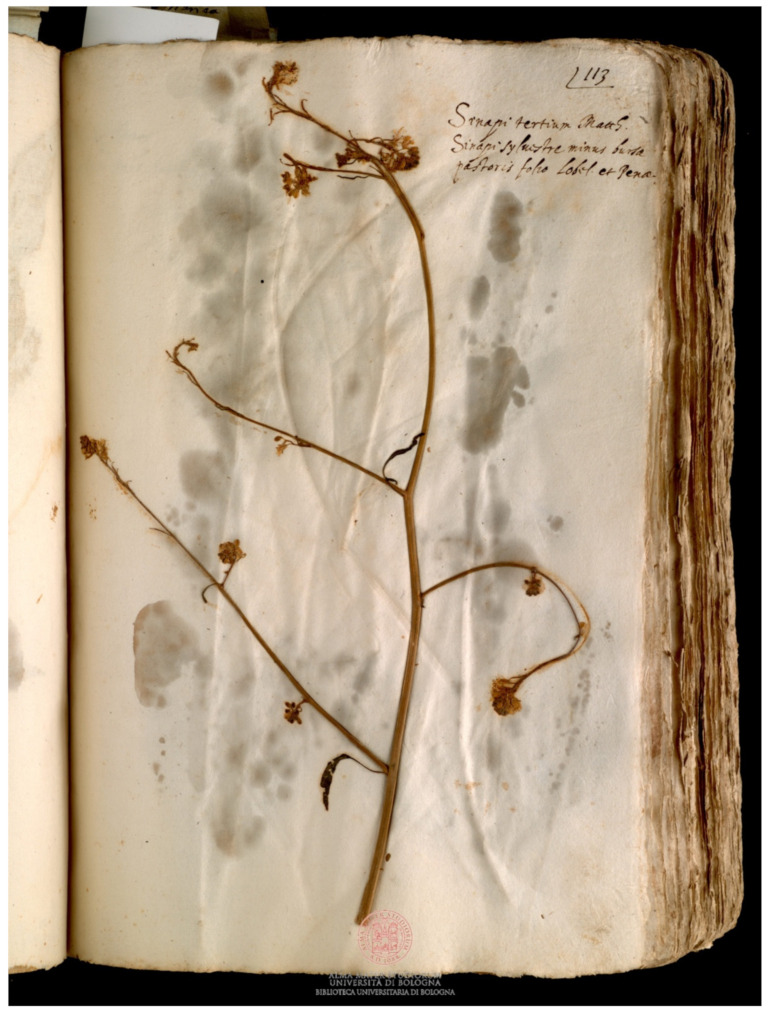
Specimen of *Brassica nigra* W.D.J. Koch preserved in Erbario Aldrovandi, vol. II, c. 113r.; on the sheet, “Sinapi tertium Matth., Sinapi syluestre minus bursæ pastoris folio Lobel. et Penæ” is written. COPYRIGHT © Università di Bologna/Sistema Museale di Ateneo—Erbario e Orto Botanico.

**Figure 3:**
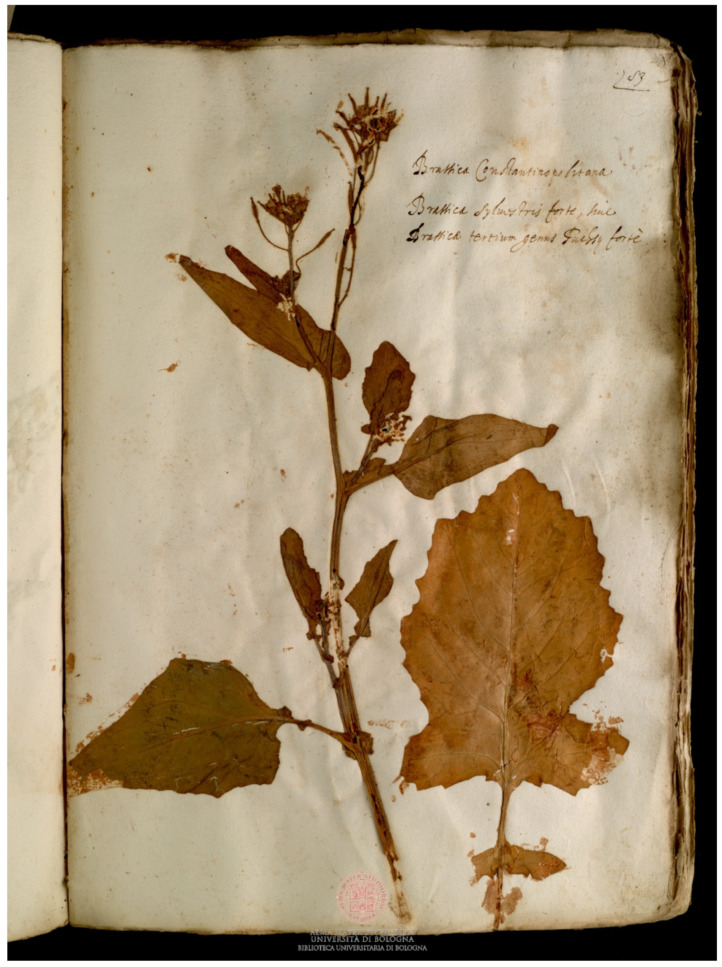
Specimen of *Brassica rapa* L. preserved in Erbario Aldrovandi, vol. V, c. 83r.; on the sheet, “Brassica Constantinopolitana, Brassica syluestris forte, siue Brassica tertium genus Fuchsij forte” is written. COPYRIGHT © Università di Bologna/Sistema Museale di Ateneo—Erbario e Orto Botanico.

**Figure 4:**
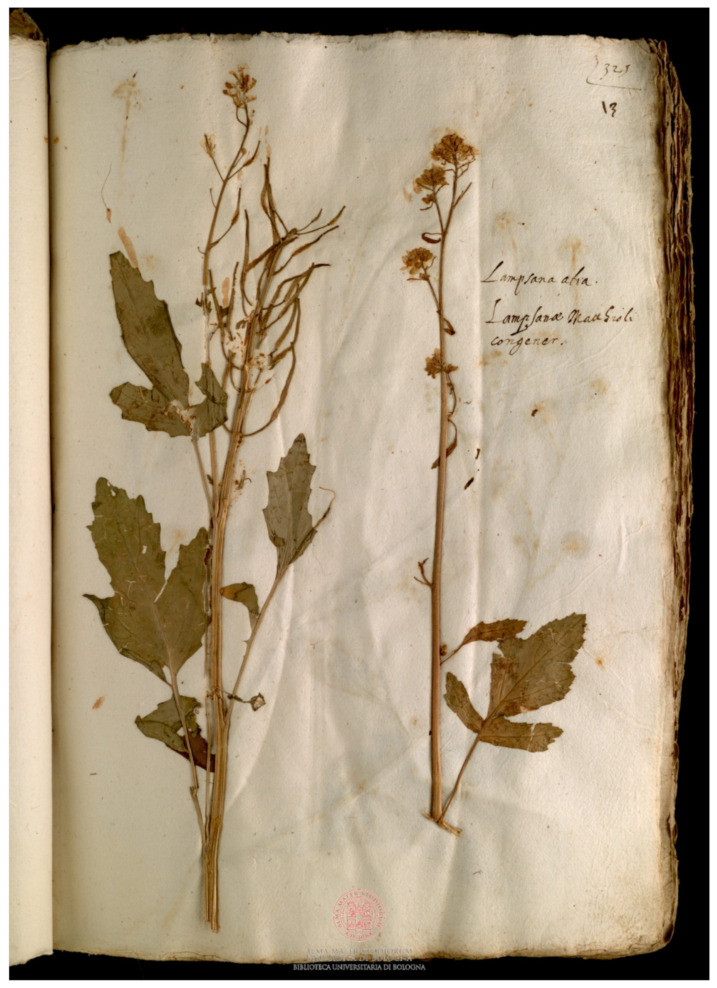
Specimen of *Sinapis alba* L. preserved in Erbario Aldrovandi, vol. IV, c. 13r.; on the sheet, “Lampsana alia, Lampsanæ Matthioli congener” is written. COPYRIGHT © Università di Bologna/Sistema Museale di Ateneo—Erbario e Orto Botanico.

*Brassica oleracea* was the most abundant species (6 specimens in Erbario Aldrovandi, 1 specimen in Erbario ex Cibo B); *B. nigra* (Figure 2) was the sole species present in all the herbaria. *B. napus* was present only in Erbario ex Cibo B (in Erbario Aldrovandi its presence was doubtful) and *B. rapa* was present only in Erbario Aldrovandi (Figure 3). *Sinapis alba* was a marginal presence, with only 3 specimens, of which 3 were in Erbario ex Cibo B and 1 was in Erbario Aldrovandi (Figure 4).

## 3. Discussion

Our archaeobotanical analyses of the seed and fruit morphology were internally consistent and suggestive of the presence of several taxa belonging to the Brassicaceae. Notably, the presence of *Sinapis alba* was found to be recognizable among the samples in which the external seed tegument was well preserved [15]. Perhaps the most interesting aspect of these data was the presence of several species of *Brassica*. The large quantity of seeds available for this genus [50] in the two contexts examined allowed the unequivocal separation of the samples into morphological groups associated with species descriptions. On this basis, the dominant species among our sites seems to have been *B. rapa* s.l., with representatives of *B. nigra* and *B. napus* also well represented. However, several studies have reported extensive intraspecific variability in the key diagnostic features of seed-surface architecture, seed size, and seed shape for species belonging to this genus [51,52,53,54]. Despite this known variability, none of the widely used diagnostic keys attempt to estimate the frequency of erroneous diagnosis [15,50,55,56,57]. We therefore sought to support identifications based on seed morphology through the use of DNA sequencing as an independent mode of species diagnosis. DNA yields from ancient biological samples (aDNA) are typically low and highly degraded [58]. However, several groups have successfully used the DNA extracted from ancient plant material to confirm species identity using short amplicons of the core DNA barcodes [59,60] or else elected to target supplementary or non-coding barcode markers [61]. Such works typically use either large volumes of starting materials [62] or next-generation sequencing platforms [63]. Currently, we are unaware of any study using aDNA for the identification of individual *Brassica* seed samples. Here, we were not able to secure high-quality barcode sequences in either *rbcL* or *matK* by direct PCR, but we were able to do so using a modified form of nested PCR [64]. Several other works have reported similar success in recovering Sanger sequence data from materials containing low levels of template DNA, including DNA from animal feces [65], adulterated food [66], formalin-preserved specimens [67], and dried museum specimens [68].

In this study, we found that our ability to use aDNA-derived sequences to differentiate between members of *Brassica* varied considerably between amplicons, with the best results deriving from the *matK*5 primer set. Reference to the seed morphology allowed us to discount all barcode-based diagnoses that fell outside the genus *Brassica*, most of which were relatively short sequence reads. It is perhaps notable that the finest level of taxonomic match was achieved after searches were performed using NCBI BLASTn searches. This may be at least partly attributable to the fact that the NCBI database contains a far higher proportion of data originating from next-generation sequencing than from Sanger sequencing, and the former is noted for its lower levels of technical errors [69]. Considering only the search results that were deemed reliable (i.e., those within *Brassica*) provided some support for the morphological identifications. Specifically, there was evidence of the significant presence of *B. nigra* in both sites, as well as of *B. oleracea*. The inability of the *matK*5 sequences to distinguish between *B. napus* and one of its progenitor species, *B. rapa* [5], precluded the confirmation of the distributions of these individual species, but was nevertheless congruent with the overall inferences of the presence made from the morphological analyses.

Both sources of plant diagnosis suggested a significant presence of *B. nigra*. The efficacy of morphological diagnosis partly depends on the characteristics of the seeds of this species. In fact, in the genus *Brassica*, originally, dark-seeded species (e.g., *B. nigra* and *B. napus*) and yellow-seeded species (e.g., *B. rapa* and *B. juncea*) are present [70]: the first have significantly more lignin than the latter [71], especially in the inner epidermis of the seed-coat (testa) [72,73], perhaps also because the seeds of *B. nigra* must undergo a necessary dormancy period before germination [72]. This feature surely makes the seeds of *B. nigra* more resistant than others, and the careful choice of seeds made in view of the aDNA analyses probably caused a slight over-representation of this taxon.

The total number of specimens found in the Renaissance herbaria was not high if considered in the light of the economic importance of these species, and the presence of samples was limited to the three herbaria richest in *exsiccata* (Erbario Aldrovandi, Erbario ex Cibo B and Erbario Cesalpino). The *B. oleracea* was probably more easily available than the others due to its widespread cultivation as a vegetable; in fact, featured 7 specimens, plus one of uncertain attribution, comprising 1/3 of the total number of specimens found in the herbaria. In this regard, Mattioli [74] referred that various cabbage “species” exist, and Durante [75] noted that cabbages were cultivated and transplanted in all kitchen gardens and vineyards. By contrast, *B. napus* and *B. rapa* were only marginal presences among the herbarium samples, whereas *B. rapa* was the most abundant species among the archaeobotanical remains. It is worth noting that during the 16th century, *B. napus* was commonly known, as attested by Mattioli [74]; therefore, it was probably commonly cultivated, and various “species” of it existed [75]; *B. rapa* was also extremely common in Italy, especially in the north [74,75]. *B. nigra*, another medicinal plant [76,77], was present in all three herbaria, even if with only one sample each; finally, *Sinapis alba* also had few records, in two herbaria only, despite its recognition as the common quality of mustard, cultivated in the kitchen gardens [74,78].

### Ethnobotanical Considerations in the Possible Uses of Brassica and Sinapis Seeds

In the two pits, the significant abundance of seeds of the *Brassica* sp.pl. and *Sinapis alba* makes it possible to hypothesize a specific use of these taxa: we observed two contexts that evidently collected waste from food preparation [24,79].

It is known that, during the Middle Ages, various species of *Brassica* were cultivated in Europe to obtain oil [80,81]. During this period, in fact, for economic, cultural, and religious reasons, the use of “minor“ vegetable oils in food preparation became quite widespread and nearly dominant in several territories, principally in those that were (at least partially) outside the range of *Olea europaea* L. [82]. In particular, the oil of rapeseed (*B. rapa/campestris* var. *oleifera*), was used up to the 20th century, particularly in northern Italy [83], where the taxon was commonly cultivated, even in the mid-1500s [74,75]. On the other hand, the oil of swede (*B. napus*) was frequently used as a condiment, as a fuel for oil lamps, to make soap, and in wool crafts [75]. Thus, the numerous seeds of *B. rapa* s.l. and *B. napus* found in the archaeological contexts of Ferrara and Lugo probably represent the waste of gentle squeezing (since not many fragments are present) to obtain oil. It should be noted that they were also used as components in complex pharmaceutical preparations, such as the *theriaca*: the seeds of *B. napus* and *B. rapa* were regarded as excellent counterpoisons and, therefore, inserted in the composition of numerous antidotes [74].

On the other hand, various seeds of the genera *Brassica* and *Sinapis* are used as spices and ingredients in sauces, in particularly *B. nigra*, *B. juncea*, and *S. alba* [84,85]. In the mid-15th century Michele Savonarola, ancestor of the more famous Girolamo, as a physician of Duke Borso d’Este in Ferrara, wrote a dietetics treaty, listing and commenting on the foods that were more or less commonly present on the tables of that epoch [86]. Among various features, Savonarola notes that in Ferrara, “ogni contrada” (every neighborhood) had two or three sales counters for “senava” (mustard), which was consequently widely used in kitchens. Here, the term “senava” much probably identifies the mostarda, which in its current form was likely codified in the Middle Ages but was subsequently differentiated in diverse regional recipes [87,88]. Mostarda can be a sauce made of crushed mustard seeds only, or a very rich and complex product, with the addition of fruits (grapes or their derivatives, figs, apples, pears, quinces, blackberries, walnuts etc.) and various spices in seeds (anise, coriander, fennel, pepper etc. [88]). It is interesting to observe that nearly all these potential ingredients were found among the botanical remains of the two pits [38,79]. Mustard is considered as one of the “universally” widespread elements of the cuisine of the late Western Middle Ages [89,90]; the contemporaneous presence of *B. nigra* and *S. alba* in both in Ferrara and Lugo suggests that this product could also have been produced in both contexts.

## 4. Materials and Methods

### 4.1. Archaeological Context

The seeds considered in this study come from two sites in Ferrara and Lugo (Emilia-Romagna—Figure 1a) with two particular contexts, described below.

(I) Ferrara—Corso Porta Reno/via Vaspergolo (Figure 1b). In a key position of the medieval city, an excavation (about 300 m^2^) was carried out in 1993–1994, which exposed a stratification of the city of early medieval foundation [91]. The chronological range of the archaeobotanical material analysed was from the 10th to the 15th century AD; at the beginning, it was a zone of peri-urban kitchen gardens with wooden structures, which subsequently became part of the historical city centre with brick houses [36,37]. The excavation context that produced the greatest number of seeds of *Brassica/Sinapis* was a brickwork pit (3.5 × 1.5 × 1.4 m—Figure 1d) for domestic waste, collocated under the floor of a house and used for a few years in the middle of the 15th century. Quality and typology of the artifacts found within this tank indicate that the dump was used by an upper/middle-class family [92,93]. The pit was probably a place to discharge food-preparation waste [38,39,79].

(II) Lugo (Ravenna)—Piazza Baracca/via Magnapassi (Figure 1c). The urban area, in the center of the town, excavated in 2009, revealed a zone with productive activities dating to between 14th and 16th century. The area featured numerous wells, which were later reused to dispose of waste, and a brickwork pit (3.7 × 2.0 × 1.9 m—Figure 1e), analogous to the pit of Ferrara, whose filling dated from the 15th–16th century (this information was provided by Chiara Guarnieri—Soprintendenza Archeologia, Belle Arti e Paesaggio Bologna-Modena-Reggio Emilia-Ferrara). A significant number of *Brassica/Sinapis* seeds were also found in this context (still unpublished).

### 4.2. Archaeobotanical Analysis

Archaeobotanical analyses were performed in the Laboratory of Palynology and Palaeobotany of the University of Modena and Reggio Emilia.

Seed identification was based on keys for the Brassicaceae (Cruciferae) and, in particular, for genera *Brassica* and *Sinapis* [15,50,55,56,57].

Identification keys for the seeds of these two genera, defined as “globose, spherical or irregular in shape” [15], take into account the structure (although scar, chalaza, and the grooves of the radicle ridge do not have significant diagnostic value [50]), the shape, and the size. However, the most discriminant features are the type of reticulation, the size of the interspace, and the character of the stippling, which can be observed overall in the middle part of the seed (on the seed coat; for further details, see Table 5). For the seeds of the genus *Brassica*, “while it is not possible in all cases to identify an individual seed with certainty, it is usually possible to make a fairly reliable separation of a mixture of species” [50].

**Figure 5:**
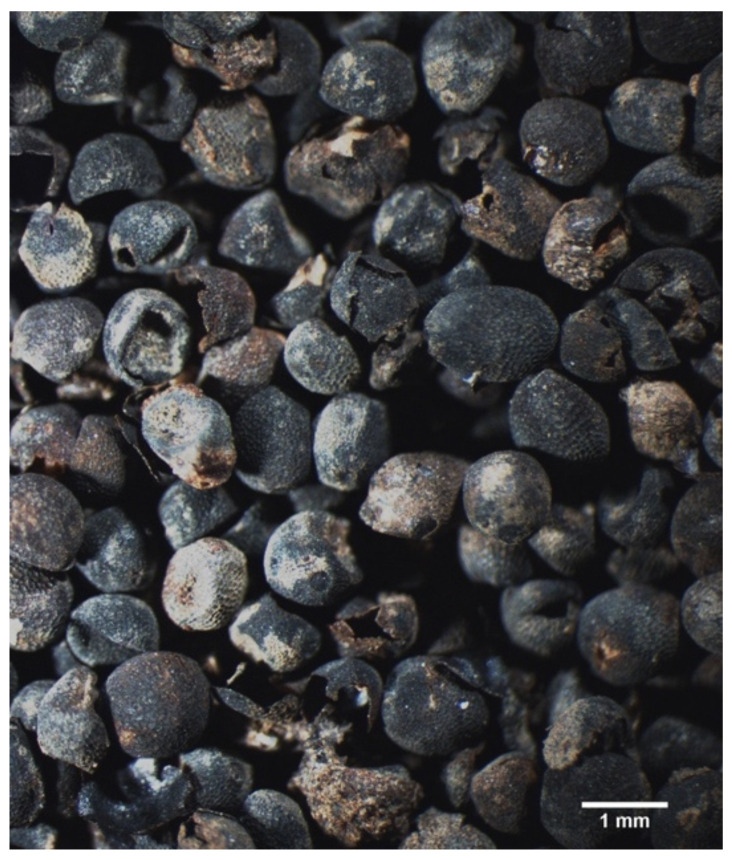
*Brassica* sp.pl. seeds from context I (Ferrara—no. 16c). Photograph: L. Dal Fiume.

Observations were made with a stereomicroscope (Figure 5) with up to 80 magnifications. Nomenclature was updated following [10,49].

### 4.3. Ancient DNA Analysis

The overall strategy for the molecular characterization of ancient seed samples was to use established chloroplast barcode markers, supplemented where necessary with chloroplast Simple Sequence Repeat (SSR) markers that were initially screened against a worldwide *Brassica* reference panel.

#### 4.3.1. Plant Materials and DNA Extraction

**Figure 6:**
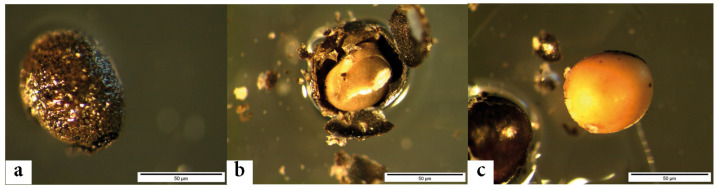
Removal of *Brassica* seed integument: (**a**) seed with integer integument, (**b**) seed with cracked integument, (**c**) seed without integument. Photographs: S. De Felice.

Archaeological seeds with a *Brassica*-type phenotype were transported to Aberystwyth, UK, for subsequent DNA extraction. Here, the following precautions were taken to minimize the probability of exogenous contamination during the extraction process. First, the seeds were not handled directly and were moved to an isolated microbiology laboratory (no previous history of plant molecular biology) for DNA extraction. Once there, seeds were initially exposed to UV light (20 min) to break and immobilize any contaminant DNA on the seed surface. Seeds were then immobilized on a sterile glass slide using nail varnish and air-dried under positive pressure. Central tissues (endosperm and embryo tissues) were then carefully removed from the seeds under sterile conditions using a dissecting microscope, and the testa (seed coats) discarded (Figure 6). The isolated internal tissues were transferred into a sterile tube containing DNeasy lysis buffer (400 μL) and RNase (20 μL) (both Qiagen, UK) and mechanically disrupted, and DNA was extracted according to the manufacturer’s instructions, except for the use of 100 μL elution buffer (rather than 50 μL).

#### 4.3.2. Primer Selection

The two universal barcode loci for plants (*rbcL* and *matK* [94]) were targeted as sites to enable species identification. For the archaeological seed samples, given the expectation of extensive degradation, a wide range of forward and reverse primers were screened to generate amplicons covering at least part of the barcoding locus (Supplementary Information, Table S2). All primers were designed using reference sequence from the BOLD database downloaded onto the Geneious software.

#### 4.3.3. Polymerase Chain Reaction of Barcoding Loci

The reaction mixture (20 μL) for each aDNA sample comprised: 1–20 ng template DNA (2–4 μL); BioMix buffer (10 μL, Bioline UK); 1 μL forward primer (1 μM), 1 μL reverse primer (1 μM); 4–6 μL nanopure water. For conventional PCR, samples were subjected to a slight variant of the following thermocycling conditions (depending on Tm values of the primers used): 94 °C (2 min), followed by 40 cycles of 94 °C (30 s), 52 °C (40 s), and 72 °C (40 s), followed in turn by a final extension at 72 °C for 10 min. For problematic materials, we used MT-PCR; a modified form of nested PCR originally described by [64] and modified by [95] was applied to problematic materials. Here, the reaction mixture (10 μL) for the preamplification comprised: 1–20 ng template DNA (1–3 μL); SensiMix buffer (5 μL, Bioline UK); 1 μL forward primer (1 μM); 1 μL reverse primer (1 μM); and 0–2 μL nanopure water. Preamplification used a slight variant of the following thermocycling conditions (depending on Tm values of the primers used): 94 °C (5 min), followed by 15 cycles of 94 °C (30 s), 52 °C (40 s), and 72 °C (40 s), followed in turn by a final extension at 72 °C for 5 min. The amplification products were first diluted 1:10 in nanopure water and then aliquoted into the following reaction mix (25 μL): SensiMix buffer (5 μL, Bioline UK); 1 μL forward primer (1 μM); 1 μL reverse primer (1 μM); and 0–2 μL nanopure water. For the selective amplification: diluted pre-amplification products (5 μL); SensiMix buffer (12.5 μL, Bioline UK); 2.5 μL of forward primer (2 μM) and reverse primer (2 μM) mix; and 5 μL nanopure water. The samples were then subjected to a minor variant of the following thermocycling regime (depending on Tm values of the primers used): 95 °C (5 min), followed by 40 cycles of 94 °C (30 s), 54 °C (40 s), and 72 °C (40 s), followed in turn by a final extension at 72 °C for 5 min.

#### 4.3.4. DNA Sequencing

Amplification products were submitted for sequence analysis to Macrogen (http://www.macrogen.com, accessed on 20 June 2012). Here, cycle-sequencing reactions were carried out according to [96]. Manual editing of raw traces and subsequent alignments of forward and reverse sequences enabled us to assign edited sequence for most species. The 3′ and 5′ termini were clipped to generate consensus sequences for each taxon. Nucleotide sequences were then translated into amino-acid sequence using ExPASY (http://www.expasy.ch/tools/dna.html, accessed on 28 November 2021).

#### 4.3.5. Sequence Analysis and Verification

Consensus sequences were produced for each taxon at each locus by alignment of the forward and reverse sequences using ClustalW (http://www.ebi.ac.uk/clustalw/, accessed on 1 December 2021). All sequences were searched on BLASTn (http://www.ncbi.nlm.nih.gov/BLAST/, accessed on 5 December 2021) or the BOLD Systems V4 database (https://www.boldsystems.org/, accessed on 5 January 2022, last visited 1 February 2022) to verify taxon (or close taxonomic group) and locus.

Following the method described here, aDNA of 242 *Brassica* seeds was analysed (Table 6): 161 from Ferrara (context I) and 81 from Lugo (context II).

### 4.4. Research in Italian Renaissance Herbaria

To compare the results of the archaeobotanical analyses with other contemporary sources, we decided to search samples of species attributable to the two genera under examination in the Italian Renaissance herbaria, the oldest in Europe (16th century—[97]); the information contained in these collections is vital to address questions related to the species or varieties cultivated and used during this epoch, especially if these data are integrated in a combined approach involving various disciplines, as already demonstrated in previous studies [98,99,100,101,102,103].

We searched *exsiccata* identified as *Brassica* or *Sinapis* species in all the Renaissance Italian herbaria (from mid-to-late 16th century): Erbario Anonimo Toscano (formerly Erbario Merini), Erbario ex Cibo B, Erbario Aldrovandi, Erbario *En Tibi*, Erbario Cesalpino, Erbario Estense. Since all of them had been extensively studied [41,42,43,44,45,46,48,104,105,106,107,108,109,110] and reliable identifications are available for nearly all the specimens, the search for samples of *Brassica* and *Sinapis* species was performed through the studies above mentioned.

## 5. Conclusions

The multiproxy approach in this research was proven to be of interest. The two waste pits of Ferrara and Lugo offered a notable and unusual quantity of seeds of *Brassica* sp.pl. and *Sinapis alba*. Thus, in this case, optimal conditions were available in which to perform the traditional morphometric analyses on the seeds in the most effective way possible, showing non-negligible species diversity within the genus *Brassica* in both contexts.

In addition, despite the aforementioned limitations, the availability of such a large volume of remains made it possible to attempt research on the aDNA for a taxon normally not studied in this sense (in contrast to the frequency with which other important economic plant species, such as *Vitis vinifera* L. or cereals, are studied [111,112,113]). Results were obtained that could form the basis for new and more in-depth investigations in this field.

Our research on the ancient herbaria (which are datable to an epoch slightly later than the contexts studied) and other historical sources of the Middle Ages/Renaissance period allowed to understand how these species were frequent and widespread among the cultivated food plants. Furthermore, the seeds of these species were used to obtain oil and other seasonings, which, from the medieval period onwards, became typical elements of all European cooking.

## Figures and Tables

**Table 1:** Brassicaceae records in Emilia-Romagna sites (6th—15th century AD). Site references for archaeobotanical analysis: 1—[23]; 2, 14, and 15—[22,24]; 3—[25]; 4—[26]; 5—[27]; 6 and 8—[28]; 7—[29]; 9—[30]; 10—[31]; 11—[32]; 12—[33,34]; 13—[35]; 16—[36,37,38,39].

Site Number		1	2	3	4	5	6	7	8	9	10	11	12		13	14	15	16		
site		Domagnano (RSM)	Rubiera (RE)	Cognento—Modena	Modena—Corso Duomo	S. Agata (BO)—Nuova Geovis	Modena—Palazzo Solmi	Parma—Piazza Garibaldi	Modena—Largo S. Francesco	Modena—ex Novi Sad	Modena -Vescovado	Forlí—ex Monte di Pietà	Argenta (FE)—via Vinarola/via Aleotti		Imola (BO)—piazza Matteotti	Ferrara—via Scandiana/San Rocco	Lugo (RA)—Piazza Baracca	Ferrara—Corso Porta Reno/via Vaspergolo		
chronology (century AD)		6th	6th–7th	end 6th–Medieval Age	7th–11th	7th–12th	10th–11th	10th–11th	10th–11th	11th–12th	12th–13th	13th–half 15th	end 13th–beginn 14th	16th	15th	15th–17th	15th–16th	second half 10th–first half 12th	13th–beginn 15th	half 14th–end 15th
context		well (Goth settlement)	hidding-well	hidding-well	layers (street)	settlement and ditch	building foundation	waste pits and cesspit	rubbish dump (city walls)	channel	ditch (Bishop’s Palace)	holes and well (for waste)	a—channel	b—cesspit (monastery Santa Caterina)	burials	hole (for waste—monastery San Vito)	brickwork pit (for waste)	a—vegetable gardens	b—urban gardens	c—brickwork pit (for waste)
*Brassica* cfr. *juncea*	seed																			X
*Brassica* cfr. *napus*	seed	X												X			X	X		XXXXX
*Brassica nigra*	seed																X			XXXXX
*Brassica* cfr. *oleracea*	seed																			XX
*Brassica rapa* s.l.	seed		X	XX				X	X		X		XX				XXXX	XXXXX	XX	XXXXXX
*Brassica* sp.	seed					X				X		X				X				XXXXX
*Sinapis alba*	seed										X						X	X		X
*Brassica/Sinapis*	seed			X	X		X	X						X	X					XXX
Brassicaceae undiff.	seed				x	x			x			x	xx		x	x	x	x		xxxxxx
*Camelina sativa*	seed																	xx		x
*Camelina* cfr. *microcarpa*	seed												x							xx
*Capsella bursa-pastoris*	seed			x		x							x					x		x
*Diplotaxis* cfr. *tenuifolia*	seed																			x
*Eruca sativa* cfr.	seed																	x		
*Isatis tinctoria*	seed																x			x
*Lepidium* sp.	seed			x	x													x		
*Myagrum perfoliatum*	silicle			xxxx	x	x	x						xx				x	x	x	
*Neslia paniculata*	silicle										x									
*Raphanus raphanistrum*	lomentum segment, seed	x											xx					x	x	x
*Rapistrum rugosum*	silicle, silicle basis				xxxx	x	x		xxxxxx	xx	xx		xxxxx			x		xxxxx	xx	xx
*Rorippa* cfr. *amphibia*	seed				x															
*Rorippa* sp.	seed																		x	x
*Sisymbrium* sp.	seed																			x
no. seeds/liter *Brassica/Sinapis*		<1	?	1	<1	<1	<1	1	<1	<1	<1	<1	1	<1	2	<1	8	13	5	1734
% *Brassica/Sinapis* seeds out of total sf		<1%	<1%	1%	<1%	<1%	<1%	<1%	<1%	<1%	<1%	<1%	<1%	<1%	<1%	<1%	<1%	1%	<1%	11%
% Brassicaceae sf out of total sf		<1%	<1%	6%	1%	<1%	<1%	<1%	7%	<1%	<1%	<1%	2%	<1%	<1%	<1%	<1%	2%	1%	11%

No. of sf: 1 to 20—x; 21 to 100—xx; 101 to 300—xxx; 301 to 500—xxxx; 501 to 1500—xxxxx; over 1500—xxxxxx.

**Table 2:** Taxonomic diagnosis using *rbcL*. Best match results recovered on BOLD Systems v4 database using amplicon sequences of *rbcL* recovered from the study sites. The percentage sequence identity and similarity score are shown, along with whether the probability of a match was lower than 10^−5^. The final column indicates the level of diagnosis possible using the sequence data alone and the species sharing the highest hit.

Sample Code	% Similarity	Score	<10^−5^	Level of Diagnosis Possible Using Sequence Alone (Top Hits in Brackets)
15–197	97.2	133	1	Spermatophyta (*Hyoscyamus niger*, *Juniperus chinesis*, *Platycladus orientalis*)
15–187	95	118	1	Spermatophyta (*Hesperotropsis leylandii*)
15–192	97	130	1	Angiospermae (*Glycine max*)
15–194	94.8	86	1	*Brassica* spp. (*Brassica rapa*, *B. oleracea* or *B. napus*)
15–199	98.5	126	1	Spermatophyta (*Picea abies*)
15–204	99.6	119	1	Angiospermae (*Orchidantha siamensis*)
15–205	\	\	\	No match > 94%
15–209	\	\	\	No match > 94%
15–201	97.5	125	0	Angiospermae (*Soleirolia soleirolii*)
16—c—145	\	\	\	No match > 94%
16—c—149	96	185	1	*Brassica* spp. (*Brassica rapa*, *B. oleracea* or *B. napus*)
16—c—151	98	189	1	Spermatophyta (*Cedrus deodara*)
16—c—152	100	201	1	*Brassica* spp. (*Brassica rapa*, *B. oleracea* or *B. napus*)
16—c—153	96	183	1	Angiospermae (*Urtica dioica*)
16—c—156	97	187	1	Angiospermae (*Glycine max*)
16—c—159	100	30	0	*Brassica* spp. (*Brassica rapa*, *B. oleracea* or *B. napus*)
16—c—159	99.1	109	1	Brassicaceae spp. (*Brassica rapa*, *B. oleracea*, *B. napus*, *Sinapis arvensis*, *Berteroa incana*, *Cardamine bulbifera*, *Cakile maritima*, *Crambe maritima*, *Sinapis alba*)
16—c—164	100	26	0	*Brassica* spp. (*Brassica rapa*, *B. oleracea* or *B. napus*)
16—c—166	97.5	110	1	Angiospermae (*Glycine max*)
16—c—167	96.7	142	1	Spermatophyta (*Hyoscyamus niger*)
16—c—169	96.6	109	1	Spermatophyta (*Hyoscyamus niger*, *Juniperus chinesis*, *Platycladus orientalis*)
16—c—171	\	\	\	Spermatophyta (no match > 94%)
16—c—172	\	\	\	Spermatophyta (no match > 94%)
16—c—173	\	\	\	Spermatophyta (no match > 94%)
16—c—174	94.1	27	0	Brassicaceae spp. (*Brassica rapa*)
16—c—181	\	\	\	Spermatophyta (no match > 94%)
16—c—182	97.8	187	1	Spermatophyta (*Pinus* spp.)
16—c—184	97	107	1	Angiospermae (*Saniculiphyllum guangxiense*)
16—c—186	98.5	197	1	*Brassica* spp. (*Brassica rapa*, *B. oleracea* or *B. napus*)
16—c—220	\	\	\	Spermatophyta (no match > 94%)
16—c—222	98	155	1	*Brassica* spp. (*Brassica rapa*, *B. oleracea* or *B. napus*)
16—c—221	97.3	671	1	Spermatophyta (*Cedrus deodara*)
16—c—121	98.4	173	1	*Brassica* spp. (*Brassica rapa*, *B. oleracea* or *B. napus*)
Control (*B. napus*)	98.3	167	1	*Brassica* spp. (*Brassica rapa*, *B. oleracea* or *B. napus*)

**Table 3:** Taxonomic diagnosis using *matK5*. Best match results recovered on BOLD Systems v4 and NCBI databases using amplicon sequences of *matK5* recovered from the study sites (only the highest match is shown). The percentage sequence identity and similarity score are shown, along with whether probability of a match was lower than 10^−5^. The species sharing the highest number of hits are provided, along with the lowest level of taxonomic diagnosis possible based on aDNA (given that all seeds possessed Brassicaceae-type seed morphology).

	Highest Sequence Match Using NCBI and BLASTn/BOLD Systems Searches				
Sample Code	% Identity	Score	e-Value	Top Hit/Hit Species/Genera	Level of Diagnosis
14–91	97.48	270	3.00 × 10^−68^	Brassicaceae spp.	Family (Brassicaceae)
15–208	99.34	276	6.00 × 10^−70^	*Brassica oleracea* (other *Brassica* spp.)	Provisional *B. oleracea*
15–253	99.18	219	8.00 × 10^−53^	*Sisymbrium aculeolatum*	Species (Brassicaceae)
15–258	100	182	8.00 × 10^−41^	*Brassica nigra*, *B. carinata*	Provisional *B. nigra*
15–267	96.86	263	5.00 × 10^−66^	*Brassica nigra*, *B. carinata* (*Diplotaxis*)	Provisional *B. nigra*
15–27	100	165	7.00 × 10^−37^	Brassicaceae spp.	Family (Brassicaceae)
15–27	100	291	2.00 × 10^−74^	*Sisymbrium aculeolatum*	Species (Brassicaceae)
15–275	100	292	6.00 × 10^−75^	*Brassica nigra*, *B. carinata* (*Diplotaxis*)	Provisional *B. nigra*
15–29	100	291	2.00 × 10^−74^	*Sisymbrium aculeolatum*	Species (Brassicaceae)
15–30	99.37	287	3.00 × 10^−73^	Brassicaceae spp.	Family (Brassicaceae)
15–31	97.48	276	6.00 × 10^−70^	Brassicaceae spp.	Family (Brassicaceae)
15–36	100	291	3.00 × 10^−74^	*Sisymbrium aculeolatum*	Species (Brassicaceae)
15–41	100	291	2.00 × 10^−74^	*Brassica napus* (*B. rapa*)	*B. napus/B. rapa*
15–88	98.09	279	5.00 × 10^−71^	Brassicaceae spp.	Family (Brassicaceae)
15–89	100	291	2.00 × 10^−74^	*Brassica napus* (*B. rapa*)	*B. napus/B. rapa*
15–90	100	291	2.00 × 10^−74^	*Brassica napus* (*B. rapa*)	*B. napus/B. rapa*
15–92	100	291	2.00 × 10^−74^	*Brassica nigra*, *B. carinata* (*Diplotaxis*)	Provisional *B. nigra*
15–93	99.36	285	1.00 × 10^−72^	Brassicaceae spp.	Family (Brassicaceae)
15–269	100	289	8.00 × 10^−74^	*Brassica nigra*, *B. carinata* (*Diplotaxis*)	Provisional *B. nigra*
15–270	100	235	8.00 × 10^−58^	*Brassica nigra*, *B. carinata* (*Diplotaxis*)	Provisional *B. nigra*
16—a—1	99.36	283	4.00 × 10^−72^	Brassicaceae spp.	Family (Brassicaceae)
16—a—1	98.31	206	5.00 × 10^−49^	*Sisymbrium aculeolatum*	Species (Brassicaceae)
16—a—10	100	198	8.00 × 10^−47^	*Sisymbrium aculeolatum*	Species (Brassicaceae)
16—a—11	98.75	141	9.00 × 10^−30^	Brassicaceae spp.	Family (Brassicaceae)
16—a—11	100	139	3.00 × 10^−29^	Brassicaceae spp.	Family (Brassicaceae)
16—a—12	100	261	1.00 × 10^−65^	*Sisymbrium aculeolatum*	Species (Brassicaceae)
16—a—14	99.08	196	3.00 × 10^−46^	Brassicaceae spp.	Family (Brassicaceae)
16—a—16	100	224	2.00 × 10^−54^	*Brassica* spp.	Genus (*Brassica*)
16—a—17	99.03	185	6.00 × 10^−43^	*Diplotaxis tenuifolia*, *Eruca vesicaria*	Family (Brassicaceae)
16—a—18	99.12	206	5.00 × 10^−49^	*Sisymbrium aculeolatum*	Species (Brassicaceae)
16—a—19	99.37	287	3.00 × 10^−73^	*Brassica oleracea* (other *Brassica* spp.)	provisional *B. oleracea*
16—a—2	100	217	3.00 × 10^−52^	*Sisymbrium aculeolatum*	Species (Brassicaceae)
16—a—20	100	291	2.00 × 10^−74^	*Brassica nigra*, *B. carinata* (*Diplotaxis*)	Provisional *B. nigra*
16—a—21	100	206	5.00 × 10^−49^	*Brassica napus* (*B. rapa*)	*B. napus/B. rapa*
16—a—223	100	281	1.00 × 10^−71^	*Diplotaxis tenuifolia*, *Eruca vesicaria*	Family (Brassicaceae)
16—a—229	100	292	6.00 × 10^−75^	*Brassica oleracea* (other *Brassica* spp.)	provisional *B. oleracea*
16—a—229	100	176	3.00 × 10^−40^	*Diplotaxis tenuifolia*, *Eruca vesicaria*	Family (Brassicaceae)
16—a—23	100	171	1.00 × 10^−38^	*Brassica* spp.	Genus (*Brassica*)
16—a—230	99.12	204	2.00 × 10^−48^	Brassicaceae spp.	Family (Brassicaceae)
16—a—231	97.44	267	4.00 × 10^−67^	*Acer* spp.	Family (non-Brassicaceae)
16—a—24	100	291	2.00 × 10^−74^	*Brassica napus* (*B. rapa*)	*B. napus/B. rapa*
16—a—3	100	263	4.00 × 10^−66^	*Brassica napus* (*B. rapa*)	*B. napus/B. rapa*
16—a—4	100	154	1.00 × 10^−33^	Brassicaceae spp.	Family (Brassicaceae)
16—a—4	100	220	2.00 × 10^−53^	Brassicaceae spp.	Family (Brassicaceae)
16—a—5	99.36	281	1.00 × 10^−71^	Brassicaceae spp.	Family (Brassicaceae)
16—a—7	100	182	7.00 × 10^−42^	Brassicaceae spp.	Family (Brassicaceae)
16—a—74	100	292	6.00 × 10^−75^	*Brassica oleracea* (other *Brassica* spp.)	Provisional *B. oleracea*
16—a—74	100	291	2.00 × 10^−74^	*Brassica oleracea* (other *Brassica* spp.)	Provisional *B. oleracea*
16—a—75	100	209	4.00 × 10^−50^	*Brassica oleracea* (other *Brassica* spp.)	Provisional *B. oleracea*
16—a—76	100	291	2.00 × 10^−74^	*Brassica oleracea* (other *Brassica* spp.)	Provisional *B. oleracea*
16—a—79	99.35	278	2.00 × 10^−70^	*Sisymbrium aculeolatum*	Species (Brassicaceae)
16—a—8	100	180	3.00 × 10^−41^	*Brassica* spp.	Genus (*Brassica*)
16—a—82	99.36	285	1.00 × 10^−72^	*Brassica nigra*, *B. carinata* (*Diplotaxis*)	Provisional *B. nigra*
16—a—83	100	291	2.00 × 10^−74^	*Sisymbrium aculeolatum*	Species (Brassicaceae)
16—a—9	100	187	2.00 × 10^−43^	Brassicaceae spp.	Family (Brassicaceae)
16—a—97	99.37	287	3.00 × 10^−73^	*Brassica nigra*, *B. carinata* (*Diplotaxis*)	Provisional *B. nigra*
16—c—121	99.34	276	6.00 × 10^−70^	*Brassica* spp.	Genus (*Brassica*)
16—c—121	99.37	287	3.00 × 10^−73^	*Brassica* spp.	Genus (*Brassica*)
16—c—123	99.35	281	1.00 × 10^−71^	*Brassica* spp.	Genus (*Brassica*)
16—c—123	97.92	165	7.00 × 10^−37^	Brassicaceae spp.	Family (Brassicaceae)
16—c—123	100	143	2.00 × 10^−30^	Brassicaceae spp.	Family (Brassicaceae)
16—c—138	100	176	3.00×10^−40^	Brassicaceae spp.	Family (Brassicaceae)
16—c—163	100	281	1.00 × 10^−71^	*Brassica nigra*, *B. carinata* (*Diplotaxis*)	Provisional *B. nigra*
16—c—163	100	292	6.00 × 10^−75^	*Brassica nigra*, *B. carinata* (*Diplotaxis*)	Provisional *B. nigra*
16—c—169	100	265	1.00 × 10^−66^	*Brassica oleracea* (other *Brassica* spp.)	Provisional *B. oleracea*
16—c—172	100	276	6.00 × 10^−70^	*Brassica oleracea* (other *Brassica* spp.)	Provisional *B. oleracea*
16—c—177	100	281	1.00 × 10^−71^	*Brassica nigra*, *B. carinata* (*Diplotaxis*)	Provisional *B. nigra*
16—c—178	100	292	6.00 × 10^−75^	*Brassica oleracea* (other *Brassica* spp.)	Provisional *B. oleracea*
16—c—247	100	292	9.00 × 10^−75^	*Brassica nigra*, *B. carinata* (*Diplotaxis*)	Provisional *B. nigra*
16—c—303	100	291	2.00 × 10^−74^	*Brassica oleracea* (other *Brassica* spp.)	Provisional *B. oleracea*
16—c—315	100	182	7.00 × 10^−42^	*Brassica* spp. (*Eruca*)	Genus (*Brassica*)
16—c—42	98.73	279	5.00 × 10^−71^	Brassicaceae spp.	Family (Brassicaceae)
16—c—45	100	291	2.00 × 10^−74^	*Sisymbrium aculeolatum*	Species (Brassicaceae)
16—c—46	99.36	287	1.00 × 10^−71^	*Brassica napus* (*B. rapa*)	*B. napus/B. rapa*
16—c—46	100	292	8.00 × 10^−75^	*Brassica napus* (*B. rapa*)	*B. napus/B. rapa*
16—c—49	98.74	281	1.00 × 10^−71^	*Brassica nigra*, *B. carinata* (*Diplotaxis*)	Provisional *B. nigra*
16—c—49	100	291	2.00 × 10^−74^	*Sisymbrium aculeolatum*	Species (Brassicaceae)
16—c—50	100	283	4.00 × 10^−72^	*Brassica napus* (*B. rapa*)	*B. napus/B. rapa*
16—c—57	100	291	2.00 × 10^−74^	*Sisymbrium aculeolatum*	Species (Brassicaceae)
16—c—59	100	285	1.00 × 10^−72^	*Brassica napus* (*B. rapa*)	*B. napus/B. rapa*
16—c—6	99.1	202	7.00 × 10^−48^	*Brassica napus* (*B. rapa*)	*B. napus/B. rapa*
16—c—62	100	291	2.00 × 10^−74^	*Brassica nigra*, *B. carinata* (*Diplotaxis*)	Provisional *B. nigra*
16—c—64	100	291	2.00 × 10^−74^	*Sisymbrium aculeolatum*	Species (Brassicaceae)
16—c—66	98.71	278	2.00 × 10^−70^	Brassicaceae spp.	Family (Brassicaceae)
16—c—67	98.73	279	5.00 × 10^−71^	*Sisymbrium aculeolatum*	Species (Brassicaceae)
16—c—69	98.74	281	1.00 × 10^−71^	*Sisymbrium aculeolatum*	Species (Brassicaceae)
16—c—70	100	291	2.00 × 10^−74^	*Sisymbrium aculeolatum*	Species (Brassicaceae)
16—c—71	99.36	287	3.00 × 10^−73^	*Brassica nigra*, *B. carinata* (*Diplotaxis*)	Provisional *B. nigra*
16—c—72	99.36	285	1.00 × 10^−72^	*Brassica nigra*, *B. carinata* (*Diplotaxis*)	Provisional *B. nigra*
16—c—73	100	291	2.00 × 10^−74^	*Sisymbrium aculeolatum*	Species (Brassicaceae)

**Table 4:** Synopsis of the specimens of *Brassica* and *Sinapis* species found in the Italian Renaissance herbaria. The identification and the attribution to a currently accepted species follow [41,42,43,44,45,46,48]; nomenclature is updated according to [49]. The question mark within brackets indicates a doubtful identification (*Brassica* cfr. *napus* etc.).

Herbaria	ex Cibo B	Aldrovandi	Cesalpino
Chronology	1550–1553	1551–1586	1555–1563
Area	Romagna? (Italy)	Italy (Bologna, Padova, Verona, Pisa), Swiss Alps, and Constantinople	Tuscany (Italy)
Depository	Biblioteca Angelica, Rome (Italy)	University of Bologna (Italy)	University of Florence (Italy)
*Brassica napus* L.	n. 831: Napus syl. qbsdam; Buniados qbsdam; Pseudobunium qbsdam	(?) vol. XIII, c. 2r: Napus satiuus. Bunias satiuus	\
	(?) n. 1157: Sinapi syl.		
*Brassica nigra* (L.) W.D.J. Koch	n. 1156: Sinapi horten.	vol. II, c. 113r: Sinapi tertium Matth., Sinapi syluestre minus bursæ pastoris folio Lobel. et Penæ	c. 194r, n. 536: Σίνηπι: Sinapis: Senapa
*Brassica oleracea* L.	n. 205: Brassica	vol. III, c. 60r: Brassica satiua, Κράμβη, Coramble Columellæ	\
		vol. III, c. 61r: Brassica arborescens Pisana	
		vol. V, c. 84r: Brassica selenites	
		vol. VIII, c. 35r: Brassica marucina folijs cœruleis, Brassica Cumana Dodon	
		vol. XIV, c. 209r: Brassica crispa Neapolitana	
		vol. XIV, c. 210r: Brassica florida, Caulfiore uulgo, Brassica nigra Dodonæi uidetur	
		(?) vol. IX, c. 119r: Brassica marucina Theoph.	
*Brassica rapa* L.	\	vol. V, c. 83r: Brassica Constantinopolitana, Brassica syluestris forte, siue Brassica tertium genus Fuchsij fortè	\
		(?) vol. VI, c. 73r: Brassicæ species	
*Brassica* sp.	\	vol. III, c. 324r: Brassica selenites seu Apiana uel crispa	\
		vol. IX, c. 135: Brassica selenites seu Apiana uel crispa	
		vol. XV, c. 61r: Brassica oris laciniosis ceu semicirculis. Brassica nigra Dodon. uidetur	
		(?) vol. I, c. 339: Napus. Navone Bonon.	
		(?) vol. VI, c. 149r: Lampsana	
		(?) vol. XIII, c. 87r: Brassica canis quibusdam	
*Sinapis alba* L.	n. 446: Erysimum aliud	vol. IV, c. 13r: Lampsana alia, Lampsanæ Matthioli congener	\
	n. 447: Erysimum aliud		

**Table 5:** Diagnostic characters used to distinguish the five principal species found during this study. Only minimum and maximum values of seed size are given.

Taxon	Species Identification Level (in This Work)	Seed Size (mm)			Reticulum Features		
		Length	Width	Thickness	Ribs	Meshes (μm)	Mesh Shape
*Brassica juncea*	cfr.	1.3–2.3	1.3–2	1.2–1.8	high and distict/conspicuos; rarely less so	100–220	elliptic
*Brassica napus*	cfr.	1.1–2.6	1.3–2.3	1.1–2.3	low and indistict	70–150	angular–elongated
*Brassica nigra*	id.	1.1–2.1	1.1–2	0.7–1.8	high and striking; rarely less so	50–150	mostly square
*Brassica oleracea*	cfr.	1.3–2.7	1.3–2.8	1.1–2.4	low and indistict	50–100	angular–oblong
*Brassica rapa*	s.l.	1.2–2.2	1.1–2.2	0.9–1.8	high and distict; rarely less so	100–150 (220)	oblong–angular
*Sinapis alba*	id.	1.8–3.1	1.8–2.8	1.5–2.4	low and indistict	30–100	\

From Dickson, C.A. *Brassica* seeds characters for Archaeobotany Workgroup, Glasgow. Z. Hazell/English Heritage and Dickson J.H. Eds., 2011, modified with Berggren [15,56].

**Table 6:** Summary of *Brassica*-type seed samples used for the aDNA analysis.

**I (no. 16c)—Ferrara—Corso Porta Reno/via Vaspergolo**	**Layer**	**No. of seeds analysed**	**No. of seeds aDNA able**	**Chronology (century)**
Analysed 161 seeds—aDNA results: 41 (25%)	1080	69	15	Mid-14th–end 15th AD
	1082	72	22	
	1095	20	4	
**II (no. 15)** **—** **Lugo (RA)** **—** **Piazza Baracca**	**Layer**	**No. of seeds analysed**	**No. of seeds aDNA able**	**Chronology (century)**
Analysed 83 seeds—aDNA results: 24 (29%)	557	4	1	15th–16th AD
	593-1	8	3	
	593-2	6	4	
	593-3	62	14	
	593-4	3	2	

## Data Availability

The Renaissance herbaria cited in the text are preserved in the herbaria of the universities of Florence (Erbario Anonimo Toscano, Erbario Cesalpino) and Bologna (Erbario Aldrovandi), in the Biblioteca Angelica of Rome (Erbario ex Cibo B), in the Naturalis Biodiversity Centre, Leiden (Erbario *En Tibi*), and in Modena State Archives (Erbario Estense).

## Supplementary Materials

The following supporting information can be downloaded at: https://www.mdpi.com/article/10.3390/plants11162100/s1. Table S1: Taxonomic diagnosis using *matK1*, *1a*, *1b*, *1c*, *4*. Best match results recovered on BOLD Systems v4 and NCBI databases using amplicon sequences of *matK1, 1a, 1b, 1c, 4* recovered from the study sites. The percentage sequence identity and similarity score shown, along with whether probability of a match was lower than 10^−5^. The species sharing the highest number of hits are provided, along with the lowest level of taxonomic diagnosis possible based on aDNA (given that all seeds possessed Brassicaceae-type seed morphology). Table S2: Sequences of barcode primers used to amplify material in this study. Target sequence from *Brassica napus* L. strain ZY036 chloroplast complete genome (Genbank: GQ861354).
