# Supplementary material for: Brassica and Sinapis Seeds in Medieval Archaeological Sites: An Example of Multiproxy Analysis for Their Identification and Ethnobotanical Interpretation
Source: Plants (Basel). 2022 Aug 12;11(16):2100. doi: 10.3390/plants11162100 (PMC9412621; doi:10.3390/plants11162100)
Supplement: Supplementary file 1 [file plants-11-02100-s001.zip › Table S2.pdf]

Supplementary Information Table S2. Sequences of barcode primers used to amplify material in this study. Primers generated from *Brassica napus* L. strain ZY036 chloroplast complete genome sequence (Genbank: GQ861354)

Primer	Sequence	Primer Position on GQ861354
*mat*K1Fo	CGAGTTCCATTCGTAATTTG	3985-4004
*mat*K1Fi	ATAGATTAGTACCTAATACGGGTA	3930-3953
*mat*K1Ro	TTATCAATATACTGCTTCTTTTAC	3809-3832
*mat*K1Ri	TACTCGTTCTTTTACACATCC	3818-3838
*mat*K1aFo	CCTAGTCCGTTTGGGTTAGGTTG	3844-3866
*mat*K1aFi	CGTTTGGGTTAGGTTGGAGATGGAT	3835-3859
*mat*K1aRo	ATCTTTAATAGGTTTCTGTTCGT	3690-3712
*mat*K1aRi	ACCTAATCGCTCTTTTGATTTTGG	3763-3786
*mat*K1bFo	TTTCCAAAATCAAAAGAGCGAT	3768-3789
*mat*K1bFi	CCAAAATCAAAAGAGCGATTAGGT	3763-3786
*mat*K1bRo	GTTCTGAAATGACATAGTGTGCG	3590-3612
*mat*K1bRi	TGAAATGACACATAGTGCGATACAGTCA	3594-3621
*mat*K1cFo	GGAGAAATTTCAAGGATATTTAGAGTTCG	3510-3538
*mat*K1cFi	GGATATTTAGAGTTCGATGGGGCTC	3501-3525
*mat*K1cRo	ATCAAACGATTCTGTTCGTACATTCG	3314-3339
*mat*K1cRi	ATTAAGCGTTTCACAATTAGTGAACTA	3344-3370
*mat*K4Fo	CGCTTAATTTTGCGAATGTACGAACAGAATC	3321-3351
*mat*K4Fi	GATTATTCCCACTAAGGATTTGAAC	3292-3316
*mat*K4Ro	CTTTTACCCGGTAACGTAGGGTTTGAACC	3027-3055
*mat*K4Ri	GTAGGGTTTGAACCAAGATTTCTAG	3042-3066
*mat*K5aFo	CTACGCAAGCAGTCTTCTCATTTACGATC	2846-2874
*mat*K5aFi	AGCAGTCTTCTCATTTACGATCGACATC	2840-2867
*mat*K5aRo	GAATGTATTTTCCATGATATCTAACATA	2697-2724
*mat*K5aRi	TTCCATGATATCTAACATAATGTAGGA	2706-2732
*mat*K5Fo	TACCGGGTAAAAGATGCCTCTT	3018-3039
*mat*K7Ro	TCATTGCACACAGCTTTCTCTATG	1797-1820
*rbc*La-f (E)	AGTCCACCGCGTAGACATTCAT	54213-54234
*rbc*L590R	ATGTCACCACAAACAGAGACT	53645-53665
*rbc*L1Fo	CTATTCCTAATTTATGTCGAGTAGACCTTG	53562-53591
*rbc*L1Fi	TGCAAGAATTCTAAATTCATGACTTGTAGGGA	53606-53637
*rbc*L1Ro	GGCTGGTAAGCCCATCGGTCCACACAGTTGTC	53842-53873
*rbc*L1Ri	CATGTACCAGTAGAAGATTCAGCAGCTACCGCAG	53808-53841
*rbc*LR319	GGTCTAAAGGGTAAGCTACATA	53942-53963
*rbc*L3Fo	ATCCGCGAAGAACTATGGTAGAGCAGTT	54184-54211
*rbc*L3Fi	TAGAGCAGTTTATGAATGTCTACGTGGTG	54202-54230

Supplementary Information Table S2 continued

Primers	Sequences 5'- 3'	Primer Position on GQ861354
*rbcL*3Ro	TCATCATTTCTTCGCATGTACCCGCAGTA	54370-54398
*rbcL*3Ri	TTCTTCGCATGTACCCGCAGTAGCATTCA	54363-54391
*rbcL*4Fo	GAGAATTGGGAGTTCCTATCGTAATGC	54417-54440
*rbcL*4Fi	TTGGGAGTTCCTATCGTAATGCATGACTACTCAAG	54422-54452
*rbcL*4Ro	TTTACCTACTACTACTGTACCCGCGTGA	54622-54641
*rbcL*4Ri	CTACTACTGTACCCGCGTGAACATGATCTC	54612-54641
*rbcL*5Fo	CGATGATTATGTTGAAAAAGACCGAAGTCTATGTT	54694-54722
*rbcL*5Fi	GAAAAAGACCGAAGTCGTGGTATC	54707-54730
*rbcL*5Ro	ATGCTTCTAGAGCTACTCGGTTAGCTACGGCA	54892-54923
*rbcL*5Ri	CTACTCGGTTAGCTACGGCACCCGGTGCATT	54881-54911
*trnH-psbA*F	ACTGCCTTGATCCACTTGGC	75-94
*trnH-psbA*R	CGAAGCTCCATCTACAAATGG	365-385
